# Characterization in Dual Activation by Oxaliplatin, a Platinum-Based Chemotherapeutic Agent of Hyperpolarization-Activated Cation and Electroporation-Induced Currents

**DOI:** 10.3390/ijms21020396

**Published:** 2020-01-08

**Authors:** Wei-Ting Chang, Zi-Han Gao, Shih-Wei Li, Ping-Yen Liu, Yi-Ching Lo, Sheng-Nan Wu

**Affiliations:** 1 Division of Cardiovascular Medicine, Chi-Mei Medical Center, Tainan 71004, Taiwan; cmcvecho2@gmail.com; 2 Department of Biotechnology, Southern Taiwan University of Science and Technology, Tainan 71004, Taiwan; 3 Institute of Clinical Medicine, College of Medicine, National Cheng Kung University, Tainan 70101, Taiwan; larry@mail.ncku.edu.tw; 4 Department of Physiology, National Cheng Kung University Medical College, Tainan 70101, Taiwan; hhelen000111tw@gmail.com (Z.-H.G.); lisway2019vic@gmail.com (S.-W.L.); 5 Division of Cardiovascular Medicine, Internal Medicine, College of Medicine, National Cheng Kung University Hospital, Tainan 70401, Taiwan; 6 Department of Pharmacology, College of Medicine, Kaohsiung Medical University, Kaohsiung 80708, Taiwan; yichlo@kmu.edu.tw; 7 Institute of Basic Medical Sciences, National Cheng Kung University Medical College, Tainan 70101, Taiwan; 8 Department of Medical Research, China Medical University Hospital, China Medical University, Taichung 40402, Taiwan

**Keywords:** pituitary cell, olfactory neuron, hyperpolarization-activated cation current, membrane electroporation-induced current, oxaliplatin

## Abstract

Oxaliplatin (OXAL) is regarded as a platinum-based anti-neoplastic agent. However, its perturbations on membrane ionic currents in neurons and neuroendocrine or endocrine cells are largely unclear, though peripheral neuropathy has been noted during its long-term administration. In this study, we investigated how the presence of OXAL and other related compounds can interact with two types of inward currents; namely, hyperpolarization-activated cation current (*I*_h_) and membrane electroporation-induced current (*I*_MEP_). OXAL increased the amplitude or activation rate constant of *I*_h_ in a concentration-dependent manner with effective EC_50_ or *K*_D_ values of 3.2 or 6.4 μM, respectively, in pituitary GH_3_ cells. The stimulation by this agent of *I*_h_ could be attenuated by subsequent addition of ivabradine, protopine, or dexmedetomidine. Cell exposure to OXAL (3 μM) resulted in an approximately 11 mV rightward shift in *I*_h_ activation along the voltage axis with minimal changes in the gating charge of the curve. The exposure to OXAL also effected an elevation in area of the voltage-dependent hysteresis elicited by long-lasting triangular ramp. Additionally, its application resulted in an increase in the amplitude of *I*_MEP_ elicited by large hyperpolarization in GH_3_ cells with an EC_50_ value of 1.3 μM. However, in the continued presence of OXAL, further addition of ivabradine, protopine, or dexmedetomidine always resulted in failure to attenuate the OXAL-induced increase of *I*_MEP_ amplitude effectively. Averaged current-voltage relation of membrane electroporation-induced current (*I*_MEP_) was altered in the presence of OXAL. In pituitary R1220 cells, OXAL-stimulated *I*_h_ remained effective. In Rolf B1.T olfactory sensory neurons, this agent was also observed to increase *I*_MEP_ in a concentration-dependent manner. In light of the findings from this study, OXAL-mediated increases of *I*_h_ and *I*_MEP_ may coincide and then synergistically act to increase the amplitude of inward currents, raising the membrane excitability of electrically excitable cells, if similar in vivo findings occur.

## 1. Introduction

Oxaliplatin (OXAL, Eloxatin^®^) belongs to a family of platinum-based chemotherapeutic compounds. In combination with 5-fluorouracil, this drug has been ever-increasingly used in the treatment of advanced colorectal or gastric cancer [1,2,3]. Despite the good safety profile, its use has been well-established to confer susceptibility to peripheral neuropathy, affecting sensory and motor nerve fibers, explaining its unsuitability for long-term treatment [1,4,5,6,7,8,9,10]. However, the ionic mechanism of OXAL-induced actions through which it effects peripheral neuropathy still remains largely unanswered.

There is growing evidence to show that the neurotoxicity of OXAL is closely connected to its modulations on functional activities of different ionic channels present in the surface membrane of electrically excitable cells [7,11,12,13,14]. For example, OXAL has been demonstrated to suppress different types of voltage-gated K^+^ or Na^+^ currents in various preparations, such as myelinated axons, sciatic-nerve preparations, and motoneuron-like cells [11,13,14,15,16]. Of interest, this agent was also recently reported to activate *I*_h_ in isolated dorsal root ganglion neurons, and this action is thought to be implicated in its modifications of pain sensation [17,18,19,20].

Hyperpolarization-activated cation current (*I*_h_) is a key determinant of repetitive electrical activity in heart cells, and in a variety of neurons, neuroendocrine, and endocrine cells [21,22,23,24,25,26,27]. This type of ionic current can conduct Na^+^ and K^+^ ions, and the current activation of its own accord can act to depolarize membrane potential, thereby adequately reaching the threshold required for action potential generation [22,28]. It is regarded to be carried by channels of the hyperpolarization-activated cyclic nucleotide-gated (HCN) gene family, named HCN1, HCN2, HCN3, and HCN4 [28]. Generation of HCN1-, HCN2-, and HCN4-deficient mice has highlighted the important role of these channels in regulation of pacemaker activity [26,29]. Of interest, previous reports have shown the ability of either MEL55A, an inhibitor of *I*_h_, or dexmedetomidine to ameliorate OXAL-induced pain sensation [18,19,30].

Membrane electroporation (MEP) applies an external electrical field to effect a considerable increase in the electrical conductivity and permeability of the plasma membrane [31,32,33]. Such a maneuver has been tailored to electrotransfer membrane-impermeant molecules, including DNAs, anti-neoplastic drugs, and antibodies, into cells’ interiors. Specifically, by applying an electrical field to the cells that just surpasses the capacitance of cell membrane, the membrane transiently becomes destabilized and permeable. Consequently, different substances can readily enter the cell. Notably, MEP-induced current (*I*_MEP_) combined with antineoplastic agents has been increasingly considered as a new therapeutic maneuver for facilitating the uptake of chemotherapeutic agents that do not easily cross the cell membrane and for the treatment of internal tumors [34,35]. However, few studies have investigated the OXAL effect on *I*_MEP_.

A recent report showed the ability of OXAL administration to elevate serum prolactin level [36]. OXAL was also reported to disrupt the endocrine axis of ACTH-cortisol and renin-angiotensin-aldosterone [37]. The treatment of OXAL is also likely to alter the functional activity of endocrine cells, including pituitary cells. However, how this compound interacts with membrane ion channels in endocrine cells remains largely unknown.

Therefore, in this study we tried to explore the effect of OXAL or other related compounds on ionic currents (e.g., *I*_h_ and *I*_MEP_) in pituitary tumor (GH_3_) cells. Whether the *I*_MEP_ in olfactory ensheathing (Rolf T1.B) cells is subject to be perturbed by OXAL was also examined. Our findings revealed that OXAL is able to stimulate both *I*_h_ and *I*_MEP_ with similar potency, which will summate to affect electrical behaviors of electrically excitable cells. These stimulatory effects presented herein tend to be acute in onset and are not thought to be mediated through the formation of platinum-DNA adducts, and they might conceivably account for pharmacological or toxicological actions of OXAL occurring in vivo.

## 2. Results

### 2.1. The Effect of OXAL on the Amplitude and Kinetics of Hyperpolarization-Activated Cation Current (I_h_) Recorded from GH_3_ Cells

**Figure 1:**
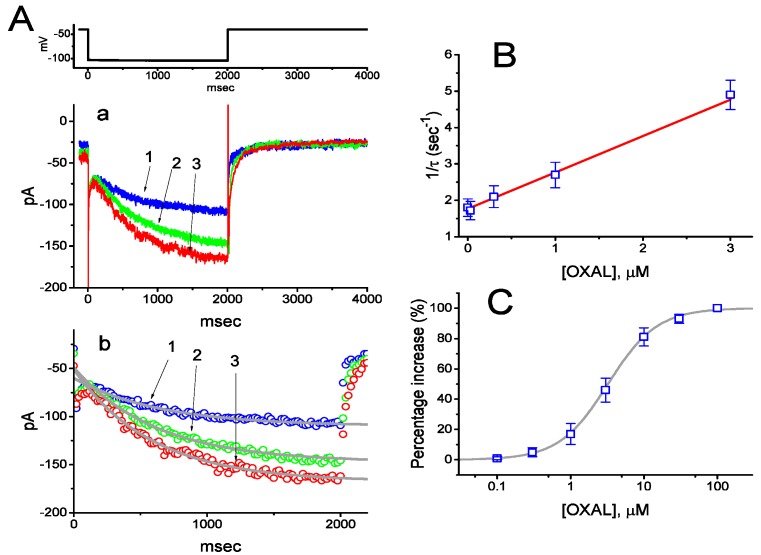
The effect of OXAL on hyperpolarization-activated cation current (*I*_h_) recorded from pituitary GH_3_ cells. (**Aa**) Representative whole-cell *I*_h_ traces obtained in the absence (1) and presence of 1 μM OXAL (2) or 0.3 μM DEX (3). The upper part in (**A**) indicates the voltage protocol used. In (**Ab**), the activation time courses of *I*_h_ taken in control (1) and during cell exposure to 1 μM OXAL (2), and 3 μM OXAL (3) were fitted by single exponentials (indicated by smooth lines) with values of 644, 612, and 585 ms, respectively. Notably, the data points for each trajectory were reduced for clarity. In (**B**), the reciprocal of activation time constant (i.e., 1/*τ*) versus the OXAL concentration was constructed and plotted. Data points shown in square circles were fitted by a linear regression, indicating that there is a molecularity of one. From minimum reaction scheme described in Materials and Methods, blocking (*k*_+1_*) and unblocking (*k*_−1_) rate constants for OXAL-induced stimulation of *I*_h_ were calculated to be 0.236 s^−1^μM^−1^ and 1.528 s^−1^, respectively. Mean ± SEM (*n* = 8–11 for each point). (**C**) Concentration-dependent stimulation of OXAL on *I*_h_ in response to membrane hyperpolarization (mean ± SEM; *n* = 9 for each point). Current amplitude was measured at the end of each hyperpolarizing pulse from −40 to −100 mV with a duration of 2 s. The continuous line overlaid onto the data points was fitted by the Hill equation, as detailed in Materials and Methods.

In the first stage of whole-cell voltage-clamp current recordings, we explored if OXAL had any perturbations on *I*_h_ in these cells. Cells were bathed in Ca^2+^-free Tyrode’s solution and the recording pipette was filled with K^+^-containing solution. The compositions of these solutions are described in Materials and Methods. The purpose for using Ca^2+^-free solution in our whole-cell recordings was to avoid possible modifications by extracellular Ca^2+^ of *I*_h_. As shown in Figure 1Aa, upon membrane hyperpolarization from −40 to −100 mV with a duration of 2 s, the cation inward current with a slowly activating time course was readily evoked. This type of hyperpolarization-activated current was sensitive to inhibition by CsCl, tramadol, and ivabradine. In agreement with previous studies described in different types of neurons, and neuroendocrine or endocrine cells, it was hence identified as an *I*_h_ [24,27,38,39]. Of interest, addition of OXAL immediately led to an increase in the amplitude of *I*_h_ in these cells. For example, as cells were exposed to 3 μM OXAL, current amplitude at the level of −100 mV significantly increased from 109 ± 18 to 168 ± 27 pA (*n* = 8, *p* < 0.05). After washout of the agent, current amplitude returned to 113 ± 22 pA (*n* = 7, *p* < 0.05). 

Apart from its reduction in *I*_h_ amplitude, the time course of *I*_h_ activation during the 2 s membrane hyperpolarization from −40 to −100 mV was noted to decrease in the presence of OXAL (Figure 1Ab). For example, addition of 3 μM OXAL produced a significant shortening of activation time constant (*τ*) of *I*_h_ from 648 ± 24 to 581 ± 21 ms (*n* = 8, *p* < 0.05). In an attempt to evaluate quantitative estimate of OXAL-mediated stimulation of *I*_h_, the values of activation time constants of *I*_h_ observed in these cells were further analyzed. The time courses for *I*_h_ activation in the presence of different OXAL concentrations were fitted using a single-exponential function by virtue of least-squares minimization procedure. The concentration dependence of *I*_h_ activation during long-step hyperpolarizing pulse is illustrated in Figure 1B. It is evident from the present observations that cell exposure to OXAL led to a concentration-dependent increase in the rate constant (i.e., 1/*τ*) of *I*_h_ activation by membrane hyperpolarization. It was also observed to display a linear relationship between 1/*τ* and the OXAL concentration with a correlation coefficient of 0.95 (Figure 1B). The forward and backward rate constants from minimum kinetic scheme detailed above in Materials and Methods were then derived and estimated to be 0.236 s^−1^μM^−1^ and 1.528 s^−1^, respectively; thereafter, the value of the dissociation constant (*K*_D_ = *k*_−1_/*k*_+1_*) turned out to be 6.47 μM. Moreover, the concentration-dependent effect of OXAL on *I*_h_ amplitude measured at the end of 2 s hyperpolarizing pulse was constructed and plotted (Figure 1C). The EC_50_ value required for OXAL-mediated stimulation of *I*_h_ seen in GH_3_ cells was calculated to be 3.2 μM, a value which is similar to the *K*_D_ value analyzed on the basis of the first-order reaction scheme.

### 2.2. Comparisons of the Effects of OXAL, OXAL Plus Ivabradine, OXAL Plus Protopine, and OXAL Plus Dexmedetomidine on I_h_ Amplitude

**Figure 2:**
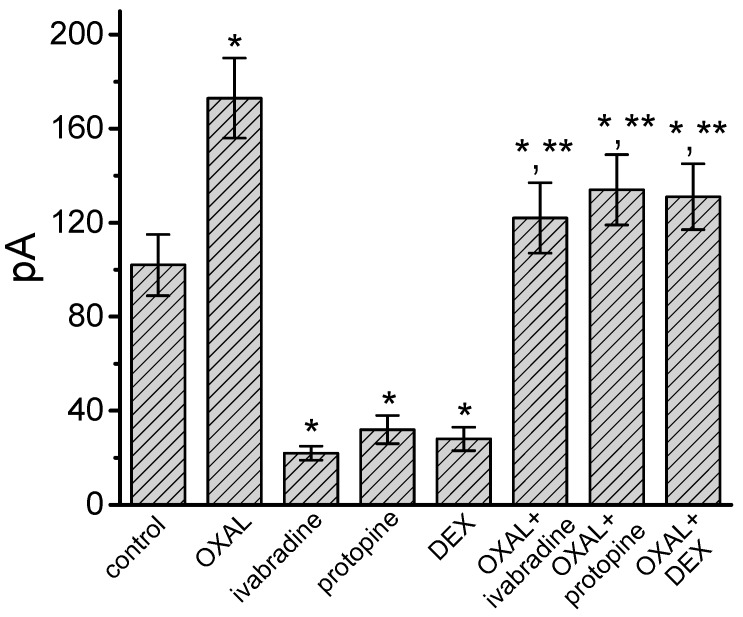
The effects of OXAL (3 μM), ivabradine (3 μM), protopine (3 μM), dexmedetomidine (DEX, 3 μM), OXAL (3 μM) plus ivabradine (3 μM), OXAL (3 μM) plus protopine (10 μM), and OXAL plus DEX (3 μM) on *I*_h_ amplitude in GH_3_ cells (mean ± SEM; *n* = 7–11 for each bar). Cells were bathed in Ca^2+^-free Tyrode’s solution and the recording pipette was filled with K^+^-containing solution. Current amplitude was measured at the end of 2-s hyperpolarizing pulse from −40 to −100 mV. * Significantly different from control (*p* < 0.05) and ** significantly different from OXAL (3 μM) alone group (*p* < 0.05).

We further examined the effects of OXAL, protopine, demedetomidine, OXAL plus ivabradine, OXAL plus protopine, and OXAL plus dexmedetomidine on the amplitude of *I*_h_ in GH_3_ cells. Similar to the OXAL action, addition of protopine (3 μM) or dexmedetomidine (3 μM) alone could suppress *I*_h_ amplitude by 69% and 73%, respectively. As depicted in Figure 2, still in the presence of 3 μM OXAL, subsequent addition of either ivabradine (3 μM), protopine (10 μM), and dexmedetomidine (3 μM) could effectively attenuate OXAL-mediated stimulation of *I*_h_ elicited by long-lasting membrane hyperpolarization. Ivabradine is an inhibitor of *I*_h_ [28,40], dexmedetomidine was previously observed to induce analgesia through a mechanism linked to the inhibition of *I*_h_ [30], and protopine was reported to suppress multiple types of ionic currents [41]. The experimental observations indicate that further addition of either ivabradine, protopine, or dexmedetomidine could attenuate OXAL-mediated increase in *I*_h_ amplitude identified in GH_3_ cells, and that the addition of protopine or dexmedetomidine alone was able to suppress *I*_h_.

### 2.3. The Effect of OXAL on the Current versus Voltage (I–V) Relationship of I_h_

**Figure 3:**
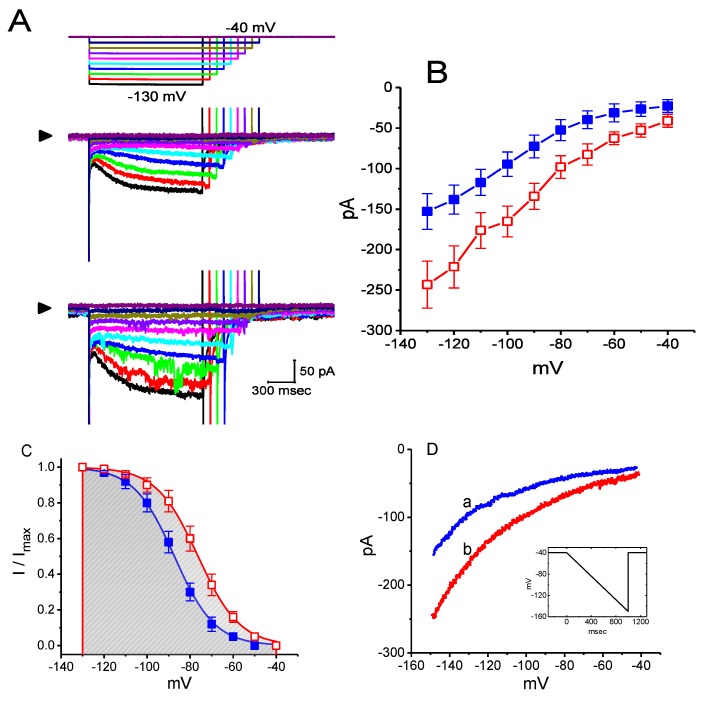
The inhibitory effects of OXAL on averaged I–V relationship of *I*_h_ measured from GH_3_ cells. In these experiments, we bathed cells in Ca^2+^-free Tyrode’s solution and filled the recording pipette by using K^+^-containing solution. (**A**) Representative whole-cell *I*_h_ traces obtained in the absence (upper part) and presence of 3 μM OXAL (lower part). Arrowhead indicates zero current level and the uppermost part in (**A**) is the voltage protocol applied. (**B**) Averaged I–V relationships of *I*_h_ measured at the end of hyperpolarizing pulses in the control (closed symbols) and during cell exposure to 3 μM OXAL (open symbols; each point indicates the mean ± SEM (*n* = 9). (**C**) The effect of OXAL (3 μM) on the steady-state activation curve of *I*_h_ (mean ± SEM; *n* = 9 for each point). ■: control; □: in the presence of 3 μM OXAL. Continuous curves were well fitted by a Boltzmann function, as described in Materials and Methods. (**D**) I–V relationship of *I*_h_ elicited in response to a long-lasting downsloping ramp pulse from −40 to −150 mV. Inset indicates the voltage protocol used. a: control; b: 3 μM OXAL.

The effects of OXAL on *I*_h_ measured at a family of hyperpolarizing steps was further studied. The averaged I–V relationships of *I*_h_ measured at the end of hyperpolarizing pulses in the control and during the exposure to 3 μM OXAL are illustrated in Figure 3A,B. Notably, from these I–V curves of the current, during the exposure to 3 μM OXAL, the macroscopic *I*_h_ conductance measured at the end of voltage steps ranging between −80 and −130 mV was significantly increased to 2.06 ± 0.05 nS (*n* = 8, *p* < 0.05) from a control value of 2.87 ± 0.08 nS (*n* = 8).

### 2.4. The Effect of OXAL on the Steady-State Activation Curve of I_h_

To characterize the inhibitory effect of OXAL on *I*_h_, we also tested if the exposure to this drug might result in the modifications on the steady-state activation curve of *I*_h_ in GH_3_ cells. Figure 3C illustrates the activation curve of *I*_h_ obtained in the absence and presence of OXAL (3 μM). Furthermore, as shown in Figure 3D, the *I*_h_ trajectories evoked in response to a 1 s downslope ramp from −40 to −150 mV with and without addition of 3 μM OXAL were examined. The observations were quite indistinguishable from the experimental results derived from those elicited by a family of maintained rectangular pulses (Figure 3B). It is, therefore, evident from the present data that the presence of OXAL exerts stimulatory action on the amplitude and gating of *I*_h_ during long-lasting membrane hyperpolarizations.

### 2.5. The Effect of OXAL on Voltage-Dependent Hysteresis of I_h_ Elicited in Response to a Long-Lasting Triangular Ramp Pulse

**Figure 4:**
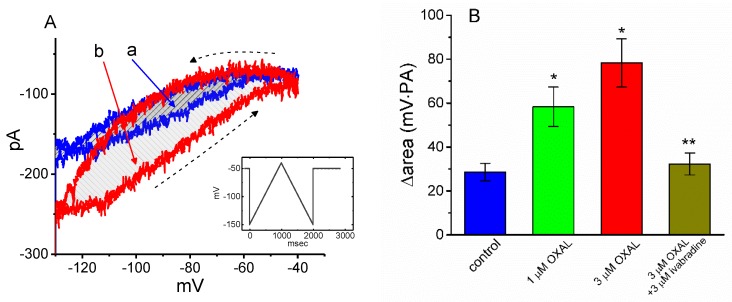
The effect of OXAL on the voltage-dependent hysteresis measured from GH_3_ cells. (**A**) Representative current trace elicited by long-lasting, 2 s triangular (i.e., upsloping and downsloping) ramp pulse between −150 and −40 mV. Insert in (**A**) is the voltage protocol applied during the recordings. (**A**) Voltage hysteresis (i.e., forward or reverse current versus voltage relationship) of *I*_h_ measured in the absence (a) and presence of 3 μM OXAL (b). Arrow depicts the direction of ramp-elicited *I*_h_ in which time passes. The current trace labeled a was the control and that labeled b was taken in the presence of 3 μM OXAL. (**B**) Summary bar graph showing the effect of OXAL and OXAL plus ivabradine on the Δarea (as indicated in shaded area in (**A**)) of voltage hysteresis (mean ± SEM; *n* = 9 for each bar). ΔArea taken with or without addition of OXAL is indicated as shaded area in (**A**). * Significantly different from control (*p* < 0.05) and ** significantly different from 3 μM OXAL alone group (*p* < 0.05).

The voltage-dependent hysteresis of *I*_h_ has been previously demonstrated to exert a high influence on electrical behaviors such as action potential (AP) firing [42,43,44]. For this reason, we further explored whether there is possible voltage-dependent hysteresis existing in *I*_h_ recorded from GH_3_ cells. In this set of experiments, we exploited a long-lasting triangular ramp pulse with a duration (i.e., 0.11 V/s) for measurement of the hysteretic properties, as whole-cell configuration was established. It is evident from Figure 4A that the trajectory of *I*_h_ elicited by the upsloping (i.e., depolarization from −150 to −40 mV) and downsloping (hyperpolarization from −40 to −150 mV) ramp pulse as a function of time was virtually distinguishable between these two limbs. The current amplitude elicited during the upsloping limb of triangular voltage ramp was higher than that by the downsloping limb; that is, there is a voltage-dependent hysteresis for this current; namely, the relationship of *I*_h_ versus membrane potential. As the ramp speed became reduced, the hysteresis degree for *I*_h_ was progressively raised. Of interest, as the examined cell was exposed to OXAL (3 μM), *I*_h_ amplitude evoked in the upsloping limb of long-lasting triangular ramp was observed to increase to a greater extent than that measured from the downsloping limb. For example, in controls, *I*_h_ values at the level of −110 mV elicited during the upsloping and downsloping limbs of triangular ramp pulse were measured to be 186 ± 19 and 123 ± 12 pA (*n* = 9), respectively, the values of which were found to differ significantly (*p* < 0.05). Specifically, as cells were exposed to 3 μM OXAL, the amplitudes of triangular ramp-induced forward and backward *I*_h_ taken at the same level of membrane potential were significantly raised to 236 ± 23 and 156 ± 17 pA (*n* = 9, *p* < 0.05), respectively.

We next quantified the degree of voltage-dependent hysteresis on the basis of the differences in areas under the curves (indicated in shaded area) in the forward (upsloping) and reverse (downsloping) directions, as described by the arrows in Figure 4B. It was seen that for *I*_h_ in GH_3_ cells, the degree of voltage hysteresis increased with slower ramp speed, and that the presence of DEX led to a conceivable reduction in the amount of such hysteresis. Figure 4B illustrates a summary of the data showing the effects of OXAL and OXAL plus ivabradine on the hysteretic area under the curve between forward and backward current traces. For example, except for the increase of *I*_h_ magnitude, addition of OXAL (3 μM) significantly elevated the area by about 1.8 fold, elicited in response to such a long-lasting triangular voltage ramp; moreover, the subsequent application of 3 μM ivabradine significantly decreased area by 60%.

### 2.6. The Effect of OXAL on Membrane Electroporation-Induced Current (I_MEP_) in GH_3_ Cells

**Figure 5:**
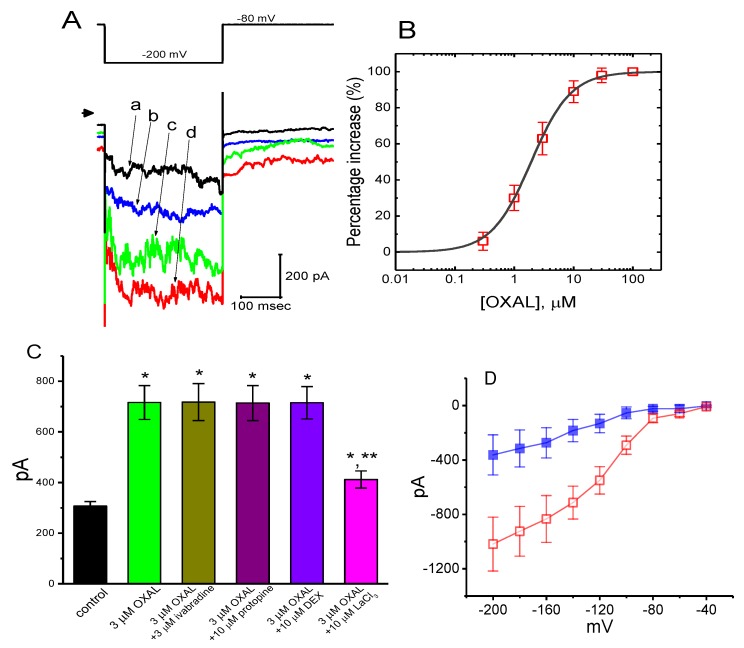
The stimulatory effect of OXAL on membrane electroporation-induced inward current (*I*_MEP_) in GH_3_ cells. Cells were immersed in Ca^2+^-free Tyrode’s solution and the examined cells were maintained at −80 mV. (**A**) Representative *I*_MEP_ traces elicited by membrane hyperpolarization from −80 to −200 mV (as indicated in the upper part). Arrowhead is zero current level; a: control; b: 1 μM OXAL; c: 3 μM OXAL; d: 10 μM OXAL. (**B**) Concentration-dependent stimulation of *I*_MEP_ by OXAL (mean ± SEM; *n* = 8 for each point). (**C**) Summary bar graph showing effects of OXAL, OXAL plus ivabradine, OXAL plus protopine, OXAL plus DEX (dexmedetomidine), and OXAL plus LaCl_3_ (mean ± SEM; *n* = 9 for each bar). Current amplitude was taken at the end of hyperpolarizing pulse from −80 to −200 mV. The smooth line represents least-squares fit to a Hill function detailed in Materials and Methods. * Significantly different from control (*p* < 0.05) and ** significantly different from 3 μM OXAL alone group (*p* < 0.05). (**D**) Averaged I–V relationships of *I*_MEP_ obtained in the absence (closed squares) and presence (open squares) of 3 μM OXAL (mean ± SEM; *n* = 9 for each point). As the whole-cell mode was firmly established, the cells were maintained at −80 mV and a family of voltage pulses ranging between −200 and −40 mV with a duration of 300 ms at increments of +20-mV were applied. Current amplitude was measured at the end of each voltage pulse.

Previous studies have demonstrated the presence of *I*_MEP_ elicited by large membrane hyperpolarization in different types of cells [31,32,33,45,46]. In another series of experiments, we raised the question of whether the presence of OXAL has possible modifications on this type of ionic current. We bathed cells in Ca^2+^-free Tyrode’s solution, and whole-cell current recordings were performed. As described in previous studies [31,45,46], when the cell was maintained at −80 mV, the hyperpolarizing pulse from −80 to −200 mV with a duration of 300 ms was applied to evoke *I*_MEP_. As shown in Figure 5A, as cells were exposed to OXAL, the amplitude of *I*_MEP_ was progressively enhanced. For example, at the level of −200 mV, the presence of 3 μM OXAL significantly increased *I*_MEP_ amplitude from 307 ± 18 to 716 ± 67 pA (*n* = 9, *p* < 0.05). After washout of the agent, current amplitude returned to 317 ± 23 pA (*n* = 7, *p* < 0.05). When K^+^ in the pipette solution was replaced with equimolar concentration of NMDG^+^, this current could still be induced by addition of 3 μM OXAL, although current amplitude was relatively smaller.

The relationship between the OXAL concentration and the percentage increase of *I*_MEP_ was constructed (Figure 5B). The half-maximal concentration required for its stimulation of *I*_MEP_ was estimated to be 1.3 μM. However, in the continued presence of 3 μM OXAL, subsequent addition of ivabradine (3 μM), protopine (10 μM), or dexmedetomidine (3 μM) was observed to fail to attenuate its stimulation of *I*_MEP_ (Figure 5C). Conversely, further addition of LaCl_3_ (10 μM) reversed OXAL-induced increase in *I*_MEP_, as evidenced by a significant reduction of *I*_MEP_ to 412 ± 34 pA (*n* = 8, *p* < 0.05).

The averaged I–V relationship of *I*_MEP_ with or without addition of OXAL is illustrated in Figure 5D. Notably, following the application of 3 μM OXAL, the amplitude of *I*_MEP_ elicited in response to membrane hyperpolarization was increased throughout the entire voltage-clamp steps examined. The threshold for elicitation of *I*_MEP_ in the control was observed to be around −100 mV, while that for current elicitation during cell exposure to 3 μM OXAL became depolarized to −80 mV. Moreover, cell exposure to OXAL (3 μM) significantly raised the slope of the linear fit of *I*_MEP_ measured at the voltages between −120 and −200 mV from 2.96 ± 0.13 to 5.75 ± 0.32 nS (*n* = 8, *p* < 0.05). Therefore, as GH_3_ cells were continuously exposed to OXAL, the I–V relationship of hyperpolarization-induced *I*_MEP_ can be modified.

### 2.7. The Stimulatory Effect of OXAL on I_h_ in Pituitary R1220 Cells

**Figure 6:**
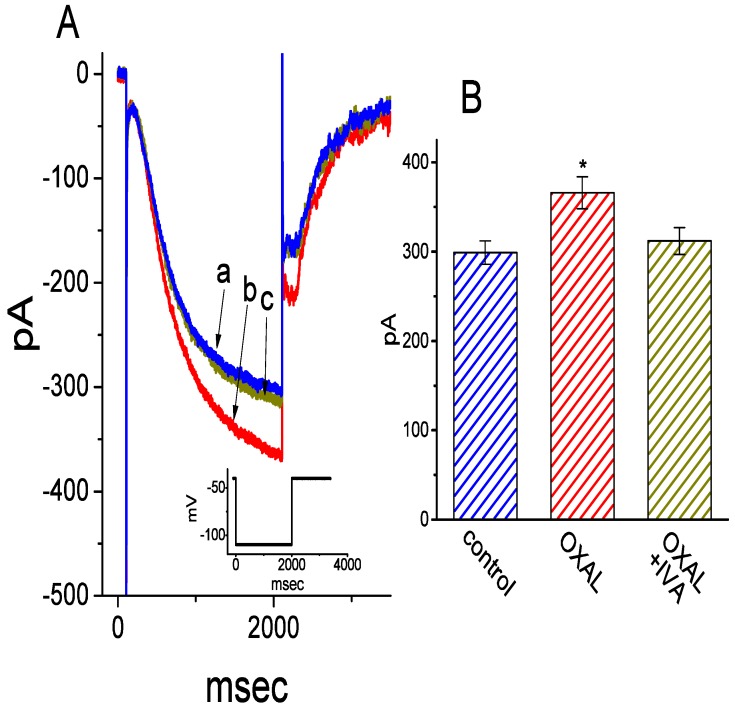
The stimulatory effect of OXAL on *I*_h_ in rat pituitary R1220 cells. Cells were immersed in Ca^2+^-free Tyrode’s solution and the examined cells were maintained at −40 mV. (**A**) Representative *I*_h_ traces elicited by a 2 s step hyperpolarization from −40 to −110 mV (as indicated in inset). a: control; b: 1 μM OXAL; c: 1 μM OXAL plus 3 μM ivabradine. (**B**) Summary bar graph showing effects of OXAL (1 μM), OXAL (1 μM) plus ivabradine (IVA, 3 μM) on *I*_h_ amplitude (mean ± SEM; *n* = 7 for each bar). Current amplitude was taken at the end of hyperpolarizing pulse from −40 to −110 mV. * Significantly different from control (*p* < 0.05).

We further wanted to test if *I*_h_ in other types of endocrine pituitary cells (e.g., pituitary R1220 cells) could be perturbed by OXAL. This type of rat pituitary cell was originally from neonatal, day-8 rats and cryopreserved in primary cultures [47]. As illustrated in Figure 6, within 2 min of exposing cells to OXAL (1 μM), the *I*_h_ amplitude during hyperpolarizing step was progressively increased, in combination with an increase in activation rate of this current. The presence of 1 μM OXAL increased current amplitude from 299 ± 13 to 366 ± 18 pA (*n* = 7, *p* < 0.05). Moreover, in the continued presence of OXAL, subsequent addition of ivabradine (3 μM) was able to attenuate OXAL-induced inhibition of *I*_h_, as evidenced by the reversal of *I*_h_ amplitude to 312 ± 15 pA (*n* = 7, *p* < 0.05). These experimental results reflect that the OXAL-mediated stimulation of *I*_h_ in this type of pituitary cell is indistinguishable from that mentioned above in GH_3_ cells.

### 2.8. Stimulatory Effect of OXAL on I_MEP_ Identified in Rolf B1.T Cells

**Figure 7:**
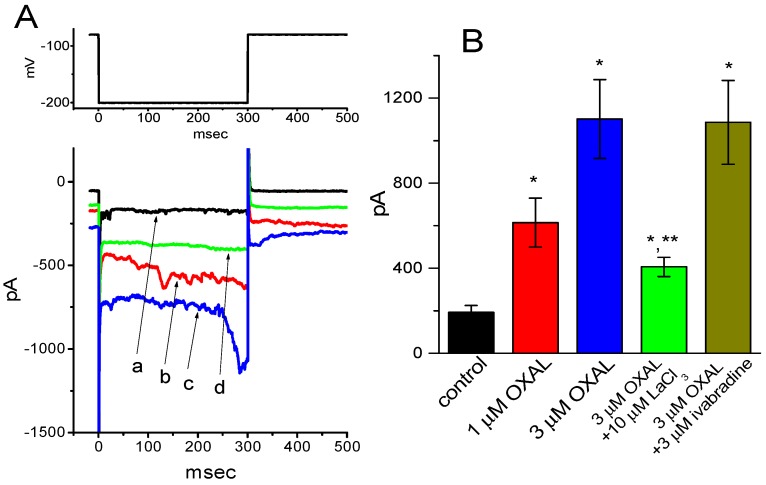
The effect of OXAL on *I*_MEP_ in Rolf B1.T olfactory ensheathing cells. In these experiments, cells were bathed in Ca^2+^-free Tyrode’s solution and the pipette was filled with K^+^-containing solution. **(A)** Representative *I*_MEP_ traces obtained in the control (a) and during the exposure to 1 μM OXAL (b), 3 μM OXAL (c), or 10 μM OXAL (d). The voltage protocol used is indicated in the upper part. Note that the presence of OXAL produces a progressive increase in *I*_MEP_. (**B**) Summary bar graph showing the effects of OXAL, OXAL plus LaCl_3_, and OXAL plus ivabradine on *I*_MEP_ in these cells (mean ± SEM; *n* = 7–8 for each bar). * Significantly different from control (*p* < 0.05) and ** significantly different from 3 μM OXAL along group (*p* < 0.05).

In a final set of recordings, we further examined if the presence of OXAL could cause any effects on *I*_MEP_ inherently in Rolf B1.T cells. The results showed that addition of OXAL effectively increased *I*_MEP_ in a concentration-dependent manner (Figure 7A). For example, as cells were exposed to 3 μM OXAL, the current measured at the level of −200 mV was significantly raised to 1102 ± 185 pA from a control of 192 ± 32 pA (*n* = 9, *p* < 0.01). Moreover, as shown in Figure 7B, subsequent addition of LaCl_3_ (10 μM), still in the presence of 3 μM OXAL, was effective at attenuating OXAL-mediated increase of *I*_MEP_ amplitude, although the inability of ivabradine to alter its stimulation of *I*_MEP_ was demonstrated in these cells. It is clear from the results that the biophysical or pharmacological properties of *I*_MEP_ are virtually distinguishable from those of *I*_h_, despite the ability of both currents to be elicited by membrane hyperpolarizations. Additionally, in accordance with the experimental observations described above in GH_3_ cells, OXAL was effective at increasing *I*_MEP_ identified in this type of olfactory neuron.

## 3. Discussion

The present study provides us with evidence showing that the presence of OXAL is able to exert dual stimulatory actions on two types of ionic currents—*I*_h_ and *I*_MEP_. It is important to note that the OXAL concentration used in this study is closely similar to that achieved in the plasma of treated patients (i.e., 3.6–5.6 μM) [48]. The stimulation by this agent of *I*_h_ seen in GH_3_ cells was not instantaneous and occurred in a time and concentration-dependent fashion, although the K_D_ value (i.e., 6.4 μM) for the stimulation of *I*_h_ tends to be greater than EC_50_ value (i.e., 3.2 μM) for its increase of *I*_h_ amplitude. Therefore, any perturbations of *I*_h_ or *I*_MEP_ induced by OXAL depend not simply on the OXAL concentration, but also on different factors, such as membrane potential, intracellular Ca^2+^ concentration, and cell volume.

In accordance with earlier studies [24,27,38,39], the present results demonstrated that pituitary GH_3_ and R1220 cells could functionally express a hyperpolarization-activated cation currents recognized as *I*_h_. There are four mammalian subtypes (HCN1, HCN2, HCN3, and HCN4) which have been cloned to date [43,49,50]. It has been demonstrated that different HCN isoforms might combine to constitute macroscopic *I*_h_ existing in different types of neurons and endocrine or neuroendocrine cells [25,38,50]. As HCN2, HCN3, or mixed HCN2 + HCN3 channels are functionally expressed in GH_3_ cells [24], OXAL-induced stimulation of *I*_h_ in native cells does not tend to be isoform specific, although our experimental results showed that OXAL could modify the amplitude and gating of *I*_h_ in GH_3_ and R1220 cells. Because of the importance of *I*_h_ (i.e., HCNx-encoded currents) in contributing to excitability and automaticity of electrically excitable cells [18,22,28,38,40,50], findings from the present results could provide novel insights into electrophysiological and pharmacological properties of OXAL or other structurally related compounds [19,20].

Previous studies have demonstrated the ability of OXAL to stimulate transient receptor potential (TRP) ankyrin channels, the activity of which could be linked to nerve injury [51]. One might thus expect that the TRP superfamily of cation channels in GH_3_ cells could be elicited by the presence of OXAL. The earlier studies have reported the presence of *I*_MEP_ in response to membrane hyperpolarization in a variety of cells including GH_3_ cells [32,33,45,46]. It is important to note that, being distinguishable from those of *I*_h_ [23,28,49], the biophysical properties of macroscopic *I*_MEP_ exhibit themselves as virtually stochastic, but obviously not deterministic, together with variable activation time course elicited by membrane hyperpolarization [31,45,46]. Therefore, it seems unlikely that *I_MEP_* stimulated by OXAL seems unlikely to be mediated by the activity of TRP or TRP-like channels.

The present observations led us to speculate that stimulation of both *I*_h_ and *I*_MEP_ caused by OXAL may account significantly for its neurological or adverse reactions [4,5,52]. A previous report showed that HCN2 is elevated after oxaliplatin-induced neuropathic pain and that HCN2-mediated pain might be mediated through the upregulation of NR2B and the activation of the CaMKII/CREB cascade in spinal neurons [19]. The effects of OXAL on membrane ionic currents described herein were noted to be rapid in onset, and they should be upstream of the formation of platinum-DNA adducts occurring inside the nucleus [4,52].

Previous studies have shown that conventional MEP might be sufficiently large to porate cytoplasmic organelles inside the cell [53]. LaCl_3_ was reported to prevent the mitochondrial morphology transition induced by chemical injury with reactive oxygen species in *Arabidopsis* [54]. It is important to speculate that OXAL-induced stimulation of MEP-induced channels presented herein contributes to its activation of mitochondrial permeability transition pore. Our results are in parallel with a scenario showing the role of mitochondrial mechanism in platinum-induced peripheral neuropathy [55,56]. It remains to be studied to what extent the presence of OXAL can enhance MEP-elicited channels which indirectly activate the intrinsic pathway to apoptotic or necrotic changes by inducing change in mitochondrial permeability transition pore.

Voltage-dependent hysteresis of *I*_h_ is thought to exhibit a substantial role in influencing the electrical behavior of electrically excitable cells such as GH_3_ cells. In agreement with previous observations [42,43,44], the *I*_h_ natively existing in GH_3_ cells was described to undergo either a hysteretic change in its voltage dependence, or a mode shift in which the voltage sensitivity in gating charge movements of the current depends on the previous state of the channel [42,43]. In this study, we also examined the possible perturbations of OXAL on such a non-equilibrium property of *I*_h_ in GH_3_ cells. Our results clearly demonstrated that the presence of this agent was capable of enlarging such hysteresis involved in the voltage-dependent elicitation of *I*_h_. However, further application of ivabradine, still in the presence of OXAL, could attenuate OXAL-mediated increase in the area of voltage-dependent hysteresis.

Membrane hyperpolarization was not produced by the operation of delayed-rectifier K^+^ currents and voltage-gated Na^+^ currents. However, from a model nerve cell system, a recent report showed the presence of hyperpolarization-mediated change in the conduction during action potential firing [57], suggesting that either *I*_h_, *I*_MEP_ or both in response to membrane hyperpolarization might have the propensity to perturb the conduction along the axon during electrical firing. In this study we were unable to observe suppressive effect of OXAL (3 μM) on proliferating GH_3_ cells. OXAL-mediated stimulation of *I*_h_ or *I*_MEP_ might not be linked to its suppression of cell growth. In pituitary R1220 cells, which have been cryopreserved in primary cultures, the presence of OXAL was also capable of increasing *I*_h_ amplitude and further addition of ivabradine reversed its stimulation of this current. Therefore, the GH_3_ cells are not the only anterior pituitary cell types to functionally express HCN channels [25,38]. However, it would be worthwhile to explore whether OXAL-mediated stimulatory effects on *I*_h_ or *I*_MEP_ could differentially occur in pituitary tumor (GH_3_) cells and in freshly isolated pituitary lactotrophs. Further studies focusing on different cell hosts, including human pituitary cells, will be required. Also, varying cellular environments to distinguish the behavior of normal cells from tumor cells will shed the light to the avidity of oxaliplatin and its interactive modes through which it exerts the effects on *I*_h_ and *I*_MEP_.

**Figure 8:**
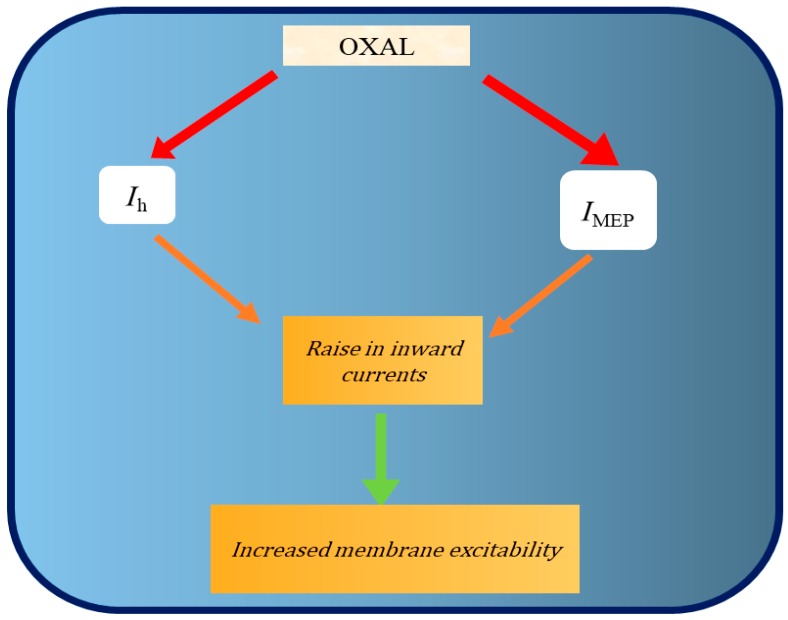
Summary of graphical representation showing consequent stimulation of OXAL on *I*_h_ and *I*_MEP_. OXAL: oxaliplatin; *I*_h_: hyperpolarization-activated cation current; *I*_MEP_: membrane electroporation-induced inward current. Red arrow indicates the stimulation of OXAL.

In agreement with several studies [7,11,13,15,16], the OXAL action in vivo is not exclusively connected to the formation of platinum-DNA adducts [4,52]. Collectively, the experimental results presented herein led us to propose that the perturbation by OXAL of *I*_h_ and *I*_MEP_ (Figure 8) is another intriguing mechanism, through the ability of it and other structurally related compounds to interfere with cell behaviors, particularly in electrically excitable cells [51], if similar in vivo findings occur.

## 4. Materials and Methods

### 4.1. Chemicals, Drugs, and Solutions

Oxaliplatin (OXAL; Eloxatin^®^, *trans*-1-diaminocyclohexane oxaliplatinum, C_8_H_14_N_2_O_4_Pt, (PubChem CID: 43805)) was acquired from Sanofi-Aventis (New York, NY, USA); ivabradine, protopine (4,6,7,14-tetrahydro-5-methylbis[1,3]benzodioxolo[4,5-c:5′,6′-*g*]azecin-13(5*H*)-one) was from Sigma-Aldrich (St. Louis, MO, USA); and dexmedetomidine (DEX) was from Abbott Laboratories (Abbott Park, IL, USA). All culture media, horse serum, fetal calf or bovine serum, *L*-glutamine, and trypsin/EDTA were obtained from Invitrogen (Carlsbad, CA, USA), unless otherwise indicated, while other chemicals, including EGTA, HEPES, LaCl_3_, aspartic acid, and *N*-methyl-D-glucamine^+^ (NMDG^+^), were of the highest purity and analytical grade.

The composition of normal Tyrode’s solution used in this study was as follows (in mM): NaCl 136.5, KCl 5.4, CaCl_2_ 1.8, MgCl_2_ 0.53, glucose 5.5, and HEPES-NaOH buffer 5.5 (pH 7.4). To record *I*_h_ or *I*_MEP_, we filled the patch electrode with the following solution (composition in mM): K-aspartate 130, KCl 20, KH_2_PO_4_ 1, MgCl_2_ 1, Na_2_ATP 3, Na_2_GTP 0.1, EGTA 0.1, and HEPES-KOH buffer 5 (pH 7.2). The medium or solution was commonly filtered using a 0.22 μm pore filter.

### 4.2. Cell Preparations

GH_3_ pituitary tumor cells, acquired from the Bioresources Collection and Research Center ((BCRC-6005); Hsinchu, Taiwan), were maintained in Ham’s F-12 medium supplemented with 15% horse serum (*v/v*), 2.5% fetal calf serum (*v/v*), and 2 mM *L*-glutamine in a humidified environment of 5% CO_2_/95% air. The clonal strain Rolf B1.T cell line was acquired from Sigma-Aldrich (lot number 03071601; St. Louis, MO, USA). This cell line was originally isolated from cultures of adult rat olfactory nerve cells. Cells have an antigenic phenotype which closely resembles that of olfactory ensheathing cells [58,59]. Rolf B1.T cells were grown in Dulbecco’s modified Eagle’s medium (Invitrogen, Carlsbad, CA, USA) supplemented with 2 mM *L*-glutamine and 10% fetal bovine serum (*v/v*). Rat pituitary cells (number R1220), which were originally isolated from neonate day-8 rats, were acquired from ScienCell Research Laboratories, Inc. (Excel Biomedical, Taipei, Taiwan), and they were grown in Epithelial Cell Medium (number 4101; ScienCell) [47,58,59]. The culture medium was refreshed every 2–3 days and cells underwent passaged when they reached confluence. Cell viability was assessed by the methylthiazole tetrazolium (MTT) salt assay. The experiments were commonly performed after 5 or 6 days of subcultivation (60–80% confluence).

### 4.3. Electrophysiological Measurements

Shortly before the experiments, cells were harvested and an aliquot of cell suspension was transferred to a home-made recording chamber mounted on the fixed stage of CKX-41 inverted microscope (Olympus, Tokyo, Japan). Cells were bathed at room temperature (20–25 °C) in normal Tyrode’s solution, the composition of which was indicated above. The recording electrodes were made of Kimax-51 glass capillaries (Kimble, Vineland, NJ, USA) by using either a PP-83 vertical puller (Narishige, Tokyo, Japan) or a P-97 Flaming/Brown horizontal puller, and their tips were then fire-polished with an MF-83 microforge (Narishige; London, UK). The electrodes used had a resistance of 3–5 MΩ and were filled with different internal solutions as detailed above. Patch-clamp whole cell recordings were performed at room temperature in standard patch-clamp technique by use of either an RK-400 amplifier (Bio-Logic, Claix, France), or an amplifier of Axopatch-200B or Axoclamp-2B (Molecular Devices, Sunnyvale, CA, USA). The junction potential between the pipette and bath solution was nulled after the pipette entered the bath but immediately before seal formation was made, and whole-cell data were hence corrected.

### 4.4. Data Recordings and Analyses

The signals, consisting of potential and current traces, were stored online on an ASUS VivoBook Flip-14 touchscreen laptop computer (TP412U; Taipei, Taiwan) at 10 kHz through a Digidata 1440A interface (Molecular Devices, Sunnyvale, CA, USA). During the experiments, the latter device was controlled by pCLAMP 10.7 (Molecular Devices). Current signals were low-pass filtered at 3 kHz with an FL-4 four-pole Bessel filter (Dagan, Minneapolis, MN, USA). The data were analyzed offline using either pCLAMP 10.7 (Molecular Devices), OriginPro (OriginLab, Northampton, MA, USA), or various custom-made macros created from Microsoft Excel^TM^ 2019.

To assess percentage increase (i.e., *y*) of OXAL on the stimulation of *I*_h_ or *I*_MEP_, the OXAL concentration required to increase 50% of current amplitude was determined using a modified Hill function in the following form:

(1) $$y=\frac{E_{max}\times {\left[\mathrm{OXAL}\right]}^{n_{H}}}{EC_{50}^{n_{H}}+{\left[\mathrm{OXAL}\right]}^{n_{H}}},$$

where [OXAL] denotes the OXAL concentration applied; n_H_ is the Hill coefficient; *EC*_50_ is the concentration required for a 50% increase, and *E*_max_ is the OXAL-maximal mediated increase in *I*_h_ or *I*_MAP_. Current amplitude during cell exposure to 100 μM OXAL was taken as 100%, and those at various concentrations of OXAL were analyzed and then compared.

The stimulatory effect of OXAL on *I*_h_ measured from GH_3_ cells is explained by using a state-dependent stimulator that preferentially binds to the open state of the HCN channel. A minimal first-order kinetic scheme was derived as follows:

(2) $$C\;\overset{\alpha }{\underset{\beta }{⇄}}\;O\;\overset{k_{+1}{}^{*}[\mathrm{OXAL}]}{\underset{{}^{{}_{k_{-1}}}}{⇄}}O\cdot \mathrm{OXAL}$$

where [OXAL] is the OXAL concentration; α and β are the voltage-gated rate constants for the opening and closing of the HCN channel, respectively; *k*_+1_^*^ and *k*_-1_ are the rate constants used for forward and backward reaction by OXAL; and C, O, and O·OXAL indicate the closed, open, and open-bound state, respectively.

The forward (i.e., on) and reverse (i.e., off) rate constants, *k*_+1_* and *k*_−1_, were determined from the activation time constants (*τ*) of *I*_h_ obtained during cell exposure to different OXAL concentrations. From the first-order reaction scheme, the rate constants collected could then be assessed using an equation of the form:

(3) $$\frac{1}{\tau }=k_{+1}^{*}\times \left[\mathrm{OXAL}\right]+k_{-1}$$

where *k*_+1_* or *k*_−1_ was respectively derived from the slope or from y-axis intercept at x-coordinate = 0 (i.e., [OXAL] = 0) of a linear function interpolating the reciprocal time constants (1/*τ*) with respect to the OXAL concentration (i.e., [OXAL]).

To characterize the stimulatory effects of OXAL on *I*_h_, the steady-state activation curve of the current with respect to command potential was constructed and compared. The relationships between the membrane potentials and the normalized amplitudes of *I*_h_ with and without addition of 3 μM OXAL were least-squares fitted with a Boltzmann function written as:

(4) $$\frac{I}{I_{max}}=\frac{1}{1+exp\left\{\frac{-\left(V-V_{\frac{1}{2}}\right)qF}{RT}\right\}},$$

where *I*_max_ denotes the maximal activated *I*_h_ or *I*_MEP_; *V* is the potential in mV; *V*_1/2_ is the membrane potential for half-maximal activation; *q* is the apparent gating charge; and *F*, *R*, and *T* is Faraday’s constant, the universal gas constant, and the absolute temperature, respectively.

### 4.5. Statistical Analyses

Curve parameter estimation was performed by a nonlinear or linear, least-squares fitting routine. We made assertions about the variability of means that could be collected from a random cohort derived from the population concerned; therefore, the standard error could have been more appropriate than the standard deviation. Data were hence presented as the means ± standard errors of the means (SEMs), with sample sizes (*n*) which indicate the number of cells collected to analyze the results, and error bars were plotted as SEMs. The paired or unpaired Student’s *t*-test or a one-way analysis of variance (ANOVA) followed by post-hoc Fisher’s least-significance difference test for multiple comparisons, were implemented for statistical analyses. The nonparametric Kruskal–Wallis test was, however, utilized, if normality underlying ANOVA tended to be violated. A probability level of <0.05 was used to define significance.
